# P-1112. Factors Associated with Symptomatic Upper Respiratory Viral Infection Symptoms and Self-COVID-19 Testing in a Multicentre Irish Healthcare Worker Cohort

**DOI:** 10.1093/ofid/ofaf695.1307

**Published:** 2026-01-11

**Authors:** Liam Townsend, Lisa Domegan, Wenzhou Wang, Siobhan Quirke, Colm Bergin, Catherine Fleming

**Affiliations:** St James's Hospital, Dublin, Ireland, Dublin, Dublin, Ireland; Health Protection Surveillance Centre, Dublin, Dublin, Ireland; Royal College of Surgeons Ireland, Dublin, Dublin, Ireland; St James's Hospital, Dublin, Ireland, Dublin, Dublin, Ireland; St. James Hospital, Dublin, Dublin, Ireland; Galway University Hospital, Galway, Galway, Ireland

## Abstract

**Background:**

Healthcare workers (HCWs) are at increased risk of viral upper respiratory tract infections (URTI) & COVID-19 compared to the general population. We utilise a longitudinal HCW study to describe symptomatic URTIs and self-testing for COVID-19 in HCWs, as well as the characteristics associated with developing URTI and subsequent COVID-19 testing.

**Figure 1:**
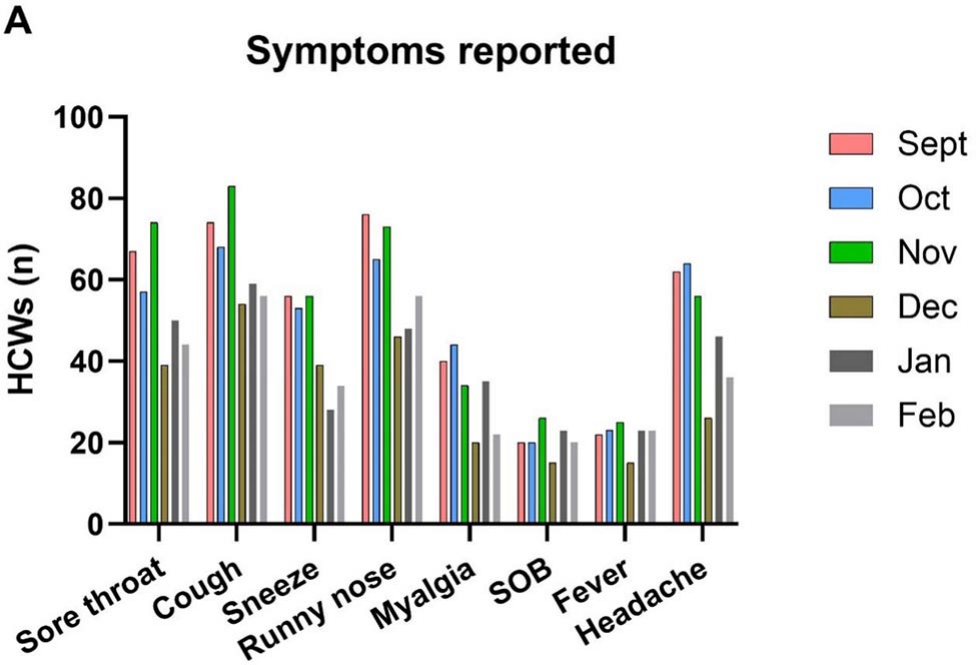
Symptom frequency across the study period

**Table 1:**
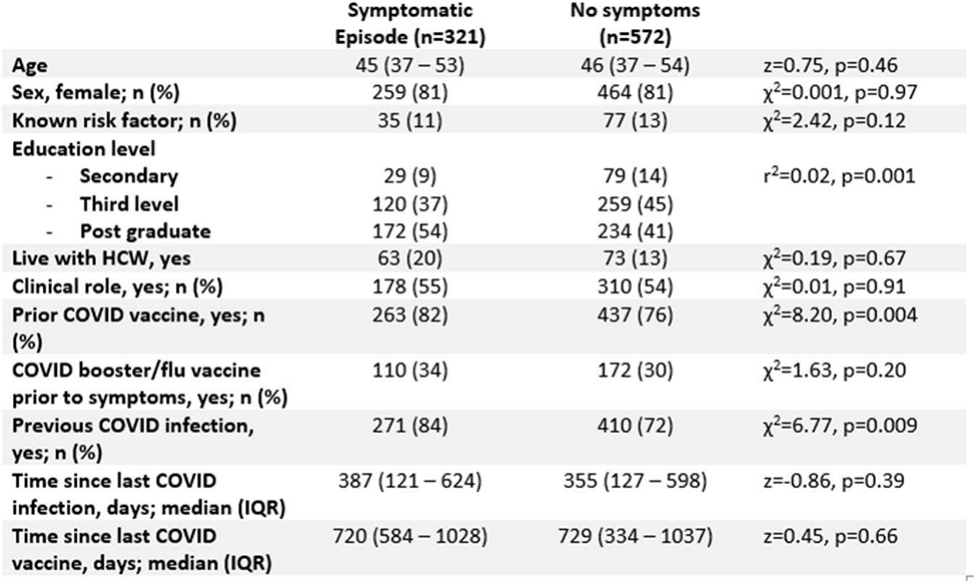
Variables associated with symptomatic illness Between-group differences assessed using Chi-squared or Wilcoxon ranksum test, as appropriate.

**Methods:**

All participants in the Prevalence of COVID-19 in Irish HCWs (PRECISE) study, a longitudinal study across two hospital sites in Ireland (St James’s Hospital, Dublin & University Hospital Galway), were included. Participants completed monthly questionnaires detailing URTI symptoms, vaccines received, and COVID-19 tests taken, as well as demographic, medical and occupation details. Data was collected from September 2024 – February 2025. Univariate analysis assessed variables associated with symptomatic illness, testing for COVID-19, and SARS-CoV-2 infection, with multivariable logistic regression including significant univariate variables and relevant variables identified *a priori*.

**Table 2:**
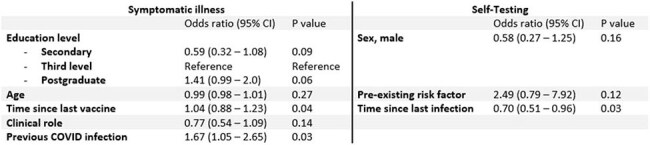
Multivariable model for factors associated with symptomatic illness and self-testing Multivariable models including significant univariate features and variables selected a priori. Time since last vaccine and time since last infection were z-scored prior to analysis.

**Table 3:**
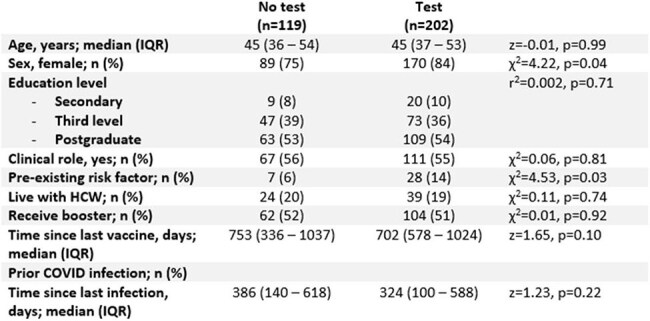
Variables associated with self-testing Between-group differences assessed using Chi-squared or Wilcoxon ranksum test, as appropriate.

**Results:**

There were n=893 participants. Symptoms suggestive of URTI were reported by n=321 (36%) (Figure 1). Symptomatic infection was more likely in participants with a prior COVID-19 infection, but independent of seasonal COVID-19 or Influenza vaccination (*Table 1*). On multivariable analysis, increasing age was associated with symptomatic episodes (*Table 2*). Of the n=321 symptomatic HCWs, 202 (63%) performed a COVID-19 antigen test. Females, those with a pre-existing risk factor for COVID infection, and shorter time since most recent COVID infection were associated with increased likelihood of testing (*Table* 3), while shorter interval since last infection was the only significant variable following multivariable regression (*Table 2*). 34 of the 202 tests (17%) were positive.

**Conclusion:**

There was a high prevalence of self-reported viral URTI symptoms among HCWs. Increasing age was associated with development of symptomatic illness. More than one-third did not perform a COVID-19 test, with shorter interval since last COVID-19 infection associated with self-testing. This study demonstrates the burden of symptomatic illness in HCWs, and the low rates of self-testing for COVID-19.

**Disclosures:**

All Authors: No reported disclosures

